# An Efficient Marker Gene Excision Strategy Based on CRISPR/Cas9-Mediated Homology-Directed Repair in Rice

**DOI:** 10.3390/ijms23031588

**Published:** 2022-01-29

**Authors:** Jiantao Tan, Yaxi Wang, Shuifu Chen, Zhansheng Lin, Yanchang Zhao, Yang Xue, Yuyu Luo, Yao-Guang Liu, Qinlong Zhu

**Affiliations:** 1 State Key Laboratory for Conservation and Utilization of Subtropical Agro-Bioresources, South China Agricultural University, Guangzhou 510642, China; tjt@scau.edu.cn (J.T.); wangyx7890@stu.scau.edu.cn (Y.W.); chenshuifu2021@126.com (S.C.); linzhansheng@stu.scau.edu.cn (Z.L.); 18320724774@stu.scau.edu.cn (Y.Z.); xueyang@stu.scau.edu.cn (Y.X.); luoyuyu@stu.scau.edu.cn (Y.L.); ygliu@scau.edu.cn (Y.-G.L.); 2 Guangdong Laboratory for Lingnan Modern Agriculture, Guangzhou 510642, China; 3 College of Life Sciences, South China Agricultural University, Guangzhou 510642, China

**Keywords:** CRISPR/Cas9, homology-directed repair, stem-, shoot tip- and inflorescence-strong promoter (P*ssi*), marker-free, rice

## Abstract

In order to separate transformed cells from non-transformed cells, antibiotic selectable marker genes are usually utilized in genetic transformation. After obtaining transgenic plants, it is often necessary to remove the marker gene from the plant genome in order to avoid regulatory issues. However, many marker-free systems are time-consuming and labor-intensive. Homology-directed repair (HDR) is a process of homologous recombination using homologous arms for efficient and precise repair of DNA double-strand breaks (DSBs). The clustered regularly interspaced short palindromic repeats (CRISPR)/CRISPR-associated protein-9 (Cas9) system is a powerful genome editing tool that can efficiently cause DSBs. Here, we isolated a rice promoter (P*ssi*) of a gene that highly expressed in stem, shoot tip and inflorescence, and established a high-efficiency sequence-excision strategy by using this P*ssi* to drive CRISPR/Cas9-mediated HDR for marker free (PssiCHMF). In our study, PssiCHMF-induced marker gene deletion was detected in 73.3% of T_0_ plants and 83.2% of T_1_ plants. A high proportion (55.6%) of homozygous marker-excised plants were obtained in T_1_ progeny. The recombinant GUS reporter-aided analysis and its sequencing of the recombinant products showed precise deletion and repair mediated by the PssiCHMF method. In conclusion, our CRISPR/Cas9-mediated HDR auto-excision method provides a time-saving and efficient strategy for removing the marker genes from transgenic plants.

## 1. Introduction

In order to effectively separate rare, transformed cells from non-transformed cells, selectable marker genes conferring antibiotic or herbicide resistance are widely employed in plant genetic transformation. However, inclusion of resistance-conferring marker genes, especially without thoroughly researching downstream ramifications, can result in public and regulatory hurdles [1]. Several strategies for obtaining transgenic marker DNA-free (marker-free) plants have been reported, such as co-transformation, homologous recombination, the transposable element system (*Ac*/*Ds*), and the site-specific recombination system (Cre/*loxP*, FLP/*FRT*, and R/*RS*) [2,3,4,5,6,7]. Among these, the Cre/*loxP* site-specific recombination system is the most widely applied auto-excision system, and uses heat-shock inducible, chemically-regulated, or tissue-specific promoters to control the expression of Cre [2,8,9,10]. The downside of these methods is that they are applicable to sexually reproducing species and thus require time-consuming breeding. Additionally, inducible Cre may result in considerable toxicity to rapidly proliferating cells [11] and a 34-bp *loxP* recombination site remains in the genome as a transgenic marker [12].

One method that has the potential to create T-DNA-free plants is homology-directed repair (HDR). HDR, a naturally occurring DNA repair system, is initiated by double-strand breaks (DSBs) and uses homologous DNA templates flanking the DSB to carry out error-free repair [13]. Homologous template arms must have a minimum length of 200–300 bp in order to induce recombination, and the efficiency is proportional to the length of the homologous region [14]. HDR offers better precision than non-homologous end joining (NHEJ), another mechanism to repair DSBs, and allows for seamless integration of DNA [13,15].

To further control the recombination process, DSBs can be induced at precise target sites using the clustered regularly interspaced short palindromic repeats (CRISPR)/CRISPR-associated nuclease Cas9 (CRISPR/Cas9). The CRISPR/Cas9 system can introduce DSBs at single or multiple target sites in the genome. CRISPR/Cas9-mediated HDR has been confirmed to be more efficient than other genome editing tools in generating gene knock-out (fragment deletion), knock-in, or allelic replacement in several organisms [16,17,18,19]. However, whether CRISPR/Cas9-mediated HDR can be employed to excise selectable marker genes, even or the entire T-DNA region, is still unknown.

Due to their multicellularity and tissue complexity, genetically manipulating plants can be more difficult than bacteria or fungi. Plants generate tissues and organs from areas of undifferentiated stem cells called meristems [20]. Through regulation of the special distribution of the hormone auxin, the shoot apical meristem (SAM) in shoot tip produces the aerial parts of the plant [21,22] and continually generates lateral organs (e.g., leaves and flowers) as the plant grows [23]. Genetic changes occurring within the SAM are transmitted to the reproductive organs and stably inherited by subsequent generations [20].

In order to address the need for a marker-free transformation method, we isolated a rice promoter (P*ssi*) that drives its gene high expression in shoot tip (containing meristem) and inflorescence (the reproductive organ of rice) and used it to drive a CRISPR/Cas9-mediated HDR (PssiCHMF) strategy in rice. This novel marker-excision tool will be useful for plant genetic engineering and molecular breeding.

## 2. Results

### 2.1. Identification of a Stem-, Shoot Tip- and Inflorescence-Strong Promoter

**Figure 1:**
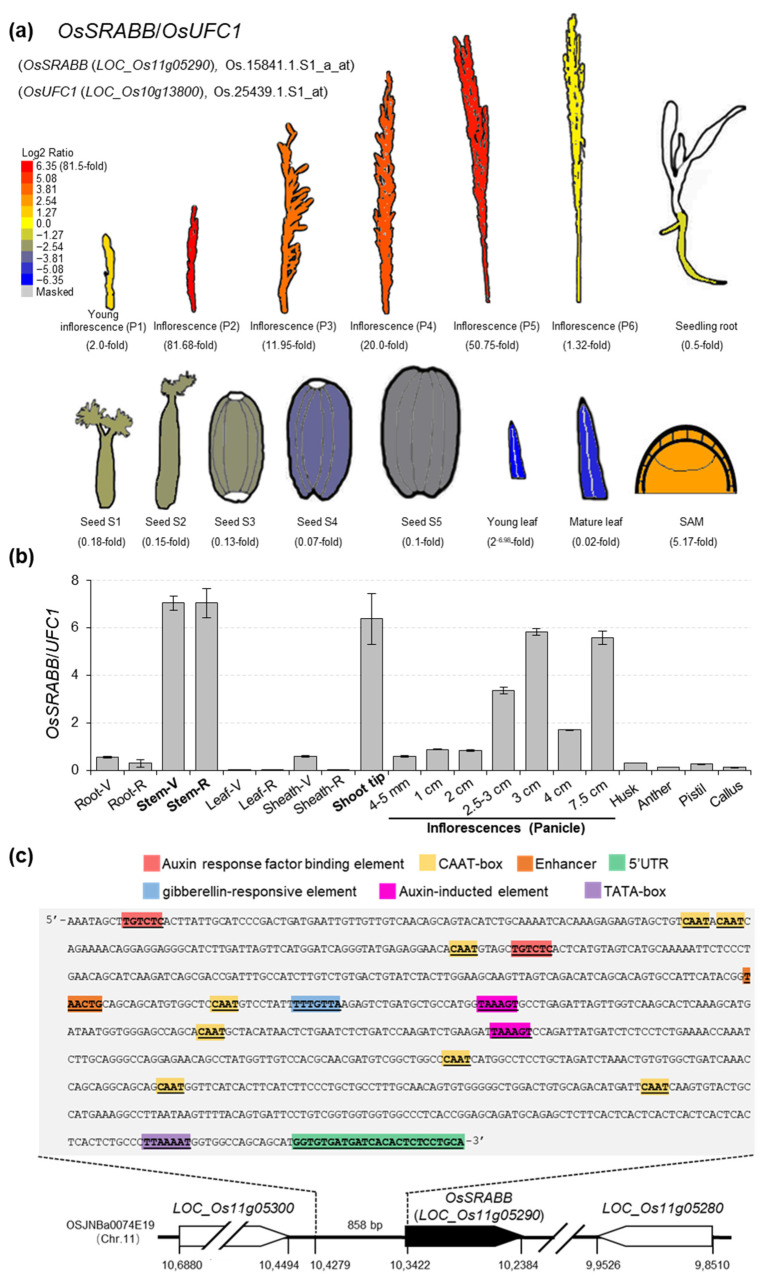
**Identification of the stem-, shoot tip- and inflorescence-strong promoter P*ssi*.** (**a**) The relative expression levels of *OsSRABB* compared with *OsUFC1* in shoot apical meristem (SAM), inflorescences, seeds, leaves and seedling root are shown as normalized data (log2) from Rice eFP Browser. Bule indicates low transcript levels, and red indicates high transcript levels. (**b**) qRT-PCR analysis of *OsSRABB* in different tissues/organs of rice (*Oryza sativa* L.) variety Zhonghua11 (ZH11). The “V” and “R” following various tissues/organs represent vegetative and reproductive stages, respectively. *OsUFC1* was used as the internal reference gene. Data are calculated from three biological replicates, and shown as mean ± SD, *n* = 3. (**c**) The distribution of *cis*-elements in different colors on the 858-bp promoter region of *OsSRABB* (P*ssi*).

In order to select an ideal promoter to drive the marker gene excision tool, we screened public transcriptomic databases Rice eFP Browser (https://bar.utoronto.ca/ (accessed on 15 December 2021)), RiceXpro (http://ricexpro.dna.affrc.go.jp/ (accessed on 15 December 2021)) and CREP (http://crep.ncpgr.cn/ (accessed on 15 December 2021)), and identified a candidate gene, *OsSRABB* (*LOC_Os11g05290*), which encodes a predicted stress responsive α/β barrel domain containing protein and is highly expressed in the SAM and inflorescences (florets) (Figure 1a) and several other tissues (such as sheath and stem) (Figure S1a), but does not express in callus (Figure S1b).

Furthermore, we found that the relative expression levels of *OsSRABB* compared with *OsActin1* (39.66-fold in SAM and 4.9–63.33-fold in inflorescences) (Figure S2) are higher than that compared with another reference gene *OsUFC1* (5.17-fold in SAM and 1.32–50.75-fold in inflorescences) in transcriptome database (Figure 1a). These results suggested that the *OsUFC1* expresses more strongly and stably than *OsActin1* in SAM and inflorescences, so we chose the *OsUFC1* as a reference gene for qRT-PCR analysis in this study. Consistent with public transcriptomic databases, expression levels of *OsSRABB* were highest in the stem, shoot tip (containing SAM) and inflorescences, especially during 2.5 to 7.5 cm of panicle (Figure 1b). These results implied that the *OsSRABB* promoter has high activity in the SAM of shoot tip and inflorescence. The promoter region of *OsSRABB* contains two auxin response factor binding elements, two auxin-responsive elements, and one gibberellin-responsive element (Figure 1c), suggesting that this promoter may respond to plant hormones and regulate gene expression. Then, we isolated an 858-bp fragment of the *OsSRABB* by PCR as a stem-, shoot tip- and inflorescence-strong promoter (P*ssi*) for further study.

### 2.2. Development of a Marker-Free Transformation Tool Based on the Pssi Driving CRISPR/Cas9-Mediated HDR

**Figure 2:**
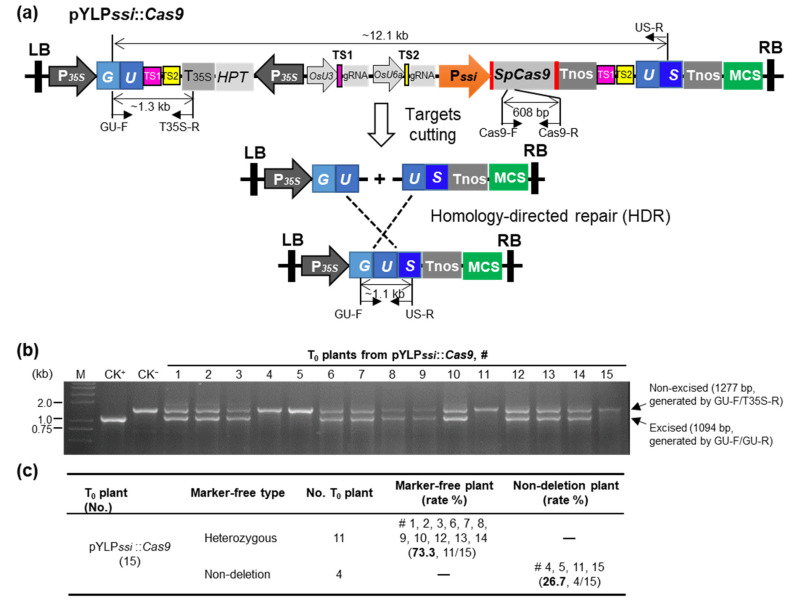
**Development of a marker-free system based onP*ssi*-driving CRISPR/Cas9-mediated homology-directed repair (PssiCHMF tool).** (**a**) Schematic diagram of the pYLP*ssi*::*Cas9* construct and the working model of the marker-free system. When CRISPR/Cas9-mediated homology-directed repair (HDR) occurs, the T-DNA carrying marker genes are excised, and the separated “*GU*” and “*US*” sequences are recombined into an intact *GUS* reporter gene. If excision is successful, the 1.1-kb recombined product is amplified by the GU-F and US-R primers, otherwise the band would be 1.3 kb, generated by GU-F and T35S-R. (**b**) PCR using primers (GU-F, T35S-R, and US-R) together to detect the excision of the marker gene cassettes in a heterozygous state (with one 1094-bp small band, generated by GU-F/US-R, and one 1277-bp large band, generated by GU-F/T35S-R) and non-excision (only with one 1277-bp large band, generated by GU-F/T35S-R) in pYLP*ssi*::*Cas9* T_0_ plants. CK^+^, pCAMBIA1305 containing intact *GUS*; CK^−^, the pYLP*ssi*::*Cas9* construct. (**c**) A summary of the efficiencies of the marker gene excision by HDR in pYLP*ssi*::*Cas9* T_0_ plants. The edited heterozygous marker-excision plants (11 plants) and non-excision plants (4 plants) are indicated.

Since the efficiency of HDR-mediated marker gene excision is an open question, we developed a marker-free transformation tool based on CRISPR/Cas9-mediated HDR (PssiCHMF: P*ssi*-driving CRISPR/Cas9-mediated homology-directed marker-free strategy) containing five main elements. First, we created a separated *GUS* expression cassette controlled by constitutive promoter P*35S*, where the GUS-encoding sequence was divided into two parts. Both parts, the front part “*GU*” and the back part “*US*”, carried a 1027-bp DNA fragment “*U*” as a homologous region for inducing HDR to achieve interval fragment deletion (Figure 2a and Figure S3). Second, two artificially designed target sites (“TS1” and “TS2”, which do not exist in the rice genome) were assembled within the flanking insides of “*GU*” and “*US*” (Figure 2a and Figure S3). Third, we created an *HPT* expression cassette for selecting resistant calli. Fourth, we created the P*ssi*-driving *SpCas9* transcriptional unit and two sgRNAs expression cassettes for TS1 and TS2, to induce DSBs during the vegetative and reproductive growth stages in meristem and inflorescence. Fifth, a fragment containing available multiple cloning sites (MCSs) was inserted outside the *GUS* expression cassette (Figure 2a and Figure S3). These elements were assembled into the engineered pCAMBIA1300 to generate the construct of pYLP*ssi*::*Cas9* for rice genetic transformation (Figure 2a). We found that, as expected, when SpCas9 endonuclease cut the target sites, the homologous arms “*U*” were exposed and recombined precisely, resulting in an intact *GUS* reporter gene (Figure 2a,b).

### 2.3. The PssiCHMF Tool Enables Efficient Excision of Marker Gene

**Figure 3:**
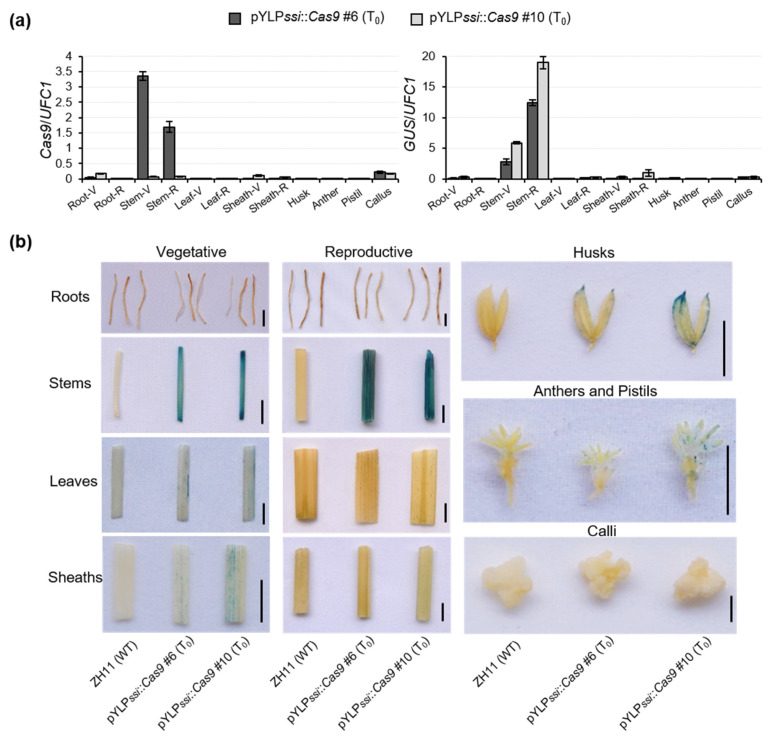
**PssiCHMF-edited recombined cells were accumulated during maturation in pYLP*ssi*::*Cas9* T_0_ plants.** (**a**) Quantification of *SpCas9* and *GUS* transcript levels in various tissues and organs of pYLP*ssi*::*Cas9* T_0_ plants. The “V” and “R” following different tissue and organ names represent vegetative and reproductive stages, respectively. *OsUFC1* was used as the internal reference gene. Data are calculated from three biological replicates, and shown as the mean ± SD, *n* = 3. (**b**) Histochemical determination of GUS activity in different tissues and organs of pYLP*ssi*::*Cas9* T_0_ plants at vegetative and/or reproductive stages. The calli were subcultured for 4 weeks. Bars = 0.5 cm.

After obtaining transgenic plants, three primers (GU-F, T35S-R and US-R, Table S1) were used to detect the HDR-mediated excision in pYLP*ssi*::*Cas9* T_0_ plants at reproductive growth stage and we used stems as samples for gDNA extraction. If the interval fragment between two homologous arms has been removed, the GU-F/US-R primers amplify a 1094-bp short band; otherwise, only a 1277-bp large product would obtain in those without fragment excision by using GU-F/T35S-R instead of the GU-F/US-R pair (~12.1-kb product, too large to amplify with short PCR cycles) (Figure 2a). The results showed that 73.3% (11/15) of the T_0_ population had successful excision of marker gene (Figure 2b,c). The above results were further proved by using a pair of primers with short (608 bp) PCR product to detect the *Cas9* expression cassette in T_0_ plant genome (Figure S4a). *SpCas9* was highly expressed in stems of T_0_ plants during the vegetative growth period, but expression dropped rapidly after reproductive growth (Figure 3a). However, *GUS* expression rose gradually from the vegetative growth period through the reproductive growth period in pYLP*ssi*::*Cas9* T_0_ plants (Figure 3a), indicating that deletions of marker fragments occur in young plants and accumulate during maturation. The number of cells containing *SpCas9* expression cassettes decreased, while those with intact *GUS* gradually increased. GUS staining was highest in stems (containing SAM) followed by husks and anthers of T_0_ plants (Figure 3b), suggesting that some recombined T-DNA-containing cells derived from the SAM have differentiated into reproductive organs, and were further enriched in inflorescences (containing husks and anthers).

### 2.4. PssiCHMF Is a Highly-Efficient and Time-Saving Marker Excision System

**Figure 4:**
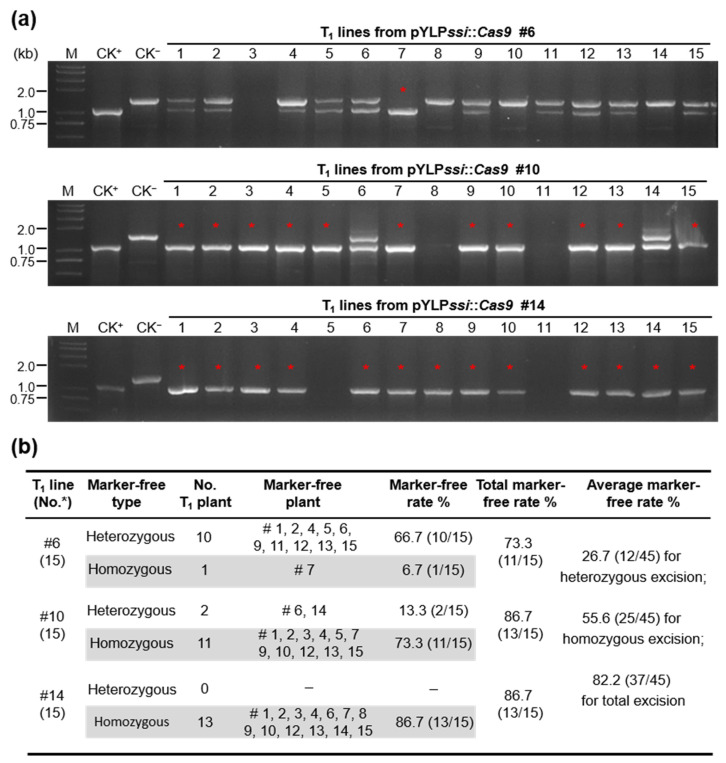
**The PssiCHMF system is a time-saving and efficient marker gene excision tool.** (**a**) Heterozygous (with two bands, generated by GU-F/T35S-R and GU-F/US-R) and homozygous (only with one small band, generated by GU-F/US-R) marker gene excision plants were confirmed in pYLP*ssi*::*Cas9* T_1_ lines by PCR to amplify the recombined products. Three independent transformed lines were used in this study. The homozygous excision plants are marked with red asterisks (*). CK^+^, pCAMBIA1305 containing intact *GUS*; CK^−^, the pYLP*ssi*::*Cas9* construct. (**b**) A summary of the efficiencies of the marker gene deletion by HDR in pYLP*ssi*::*Cas9* T_1_ lines. The edited heterozygous (average of 26.7%) and homozygous (average of 55.6%) marker-excision plants are indicated.

All three T_1_ lines derived from T_0_ plants (#6, #10, and #14) were randomly selected to detect deletions. The results showed high marker gene deletion rates (73.3%, 86.7%, and 86.7%, respectively, with an average of 82.2%), and contained both heterozygous and homozygous excision plants (Figure 4a,b). Two T_1_ lines (#10 and #14) contained a large proportion of homozygous excision (73.3% and 86.7%, respectively) (Figure 4a,b) and the *Cas9* expression cassette had been fully removed from these transgenic plants (Figure S4b), a marked improvement over other marker-free system. The Sanger sequencing of the recombinant products (the 1094-bp short band) amplified from pYLP*ssi*::*Cas9* T_1_ lines showed a seamless “*U*” fragment (Figure S5a). These results indicated that the interval fragment between the pair of “*U*” homologous arms (including the fully *HPT* expression cassette, *Cas9* expression cassette, two sgRNAs expression cassettes, the pairs of TS1 and TS2), and one of the “*U*” fragments was removed. Only an intact *GUS* gene (as a reporter for statistics of excision efficiency in this study), the T-DNA borders, and MCS region were still present in the T-DNA region.

## 3. Discussion

Meristematic tissue gives rise to all other tissues and organs in the plants. Specifically, SAM gives rise to both vegetative (e.g., leaves) and reproductive (e.g., inflorescences and florets) organs. Genetic modification of meristem (SAM) allows the transmission of modifications to flowers and seeds, and ultimately, to future generations [20]. However, because many gene-editing techniques use a resistance-conferring marker, it may be necessary to remove the marker from plant progeny to avoid unnecessary regulatory or public-relation issues. We used the stem-, shoot tip (containing SAM)- and inflorescence-strong promoter P*ssi* to drive the CRISPR/Cas9 system to produce DSBs and induce HDR, resulting in highly efficient marker gene excision (the PssiCHMF tool). GUS reporter-aided analysis of T_1_ seedlings generated from pYLP*ssi*::*Cas9* line showed constitutive expression of recombined *GUS* genes (Figure S5b), indicating that the transgenes and gene edits were indeed transmitted to the next generation. The high frequencies of marker gene homozygous excision shown in T_1_ lines further indicate that our novel marker-free system works as intended (Figure 4b).

To enable efficient HDR and remove target fragments, strong promoters are required to ensure abundant transcription of the *Cas9* nuclease encoding gene. A brief search of the public webtool funRiceGenes (https://funricegenes.github.io/ (accessed on 10 December 2021)) nets several promising results [24], such as *OsBZ1* (*LOC_Os08g28730*: brittle culm and zebra leaf I), *OsCRL6* (*LOC_Os07g31450*: crown rootless6), and *OsFON1* (*LOC_Os06g50340*: floral organ number 1). The downside to most of these promoters is that they are induced only by adverse environmental conditions, or that their constitutive expression is not high in the meristem and inflorescence [25,26,27]. Our promoter, P*ssi*, was found to drive exogenous genes highly in the meristem of stem and shoot tip, and inflorescence, rather than callus (Figure 1b and Figure 3a), ensuring that selectable markers will not be shed until the vegetative growth stage. These results indicated that P*ssi* is highly activated in the meristem of stem and shoot tip, and inflorescence, and compatible with our marker gene excision method.

HDR is a precise DSB repair pathway that allows the deletion of large DNA fragments, but it has low efficiency compared with the NHEJ pathway [17]. There are numerous methods to enhance HDR, such as chemical modulation, synchronized expression, and the use of overlapping homology arms [28,29]. The most important component of HDR-mediated genome editing is the length of the homologous regions of the repair template [17]. In our PssiCHMF system, a pair of 1027-bp homology arms was used for improving HDR efficiency, resulting in a 55.6% homozygous excision of marker genes and 82.2% total excision rate (Figure 4b). These results indicate that CRISPR/Cas9-mediated HDR is more efficient at removing marker genes than the floral- or pollen-specific promoter-controlled Cre/*loxP* system [2,10,30]. For transgenic breeding, the homologous arms should be changed to the endogenous DNA sequences of crop genome with appropriate CG content and structure, and the size of homologous region could be further shortened. In our previous work, we explored the use of microhomology-mediated end joining (MMEJ), using 3~19-bp short microhomologous sequences (MHSs), in combination with CRISPR/Cas9-based plant genomic fragment deletions [31]. Perhaps, using MMEJ, marker-free operation by the meristem- and inflorescence-strong P*ssi* also can be achieved using MHSs instead of long homology arm sequences. The T-DNA interval fragment between the pair of homologous arms would be high-efficiency auto-excision in T_0_ plants, and several homozygous excision plants would be obtained in T_1_ populations, which only kept an endogenous plant genome fragment, the T-DNA left and right borders, and MCS region in the T-DNA region, similar to T-DNA free. Further use of this promoter to replace floral-, pollen-specific or inducible promoters to drive the Cre/*loxP* marker-free system may be effective. However, the efficiency of HDR-mediated fragment deletion is highly dependent on the homologous arms, so it is necessary to further comprehensively evaluate the quality of homologous sequences.

In this study, we developed the PssiCHMF tool that allows for precise excision and seamless integration of DNA in tissues (containing meristems) with active cell growth (Figure 3b and Figure S5a). Taken together, the P*ssi*-driving CRISPR/Cas9-mediated HDR marker-free method, PssiCHMF, is a time-saving and high-efficiency marker gene removal tool capable of generating homozygous excision plants, which is a benefit for crop transgenic breeding.

## 4. Materials and Methods

### 4.1. Comprehensive Evaluation of Gene Expression, Promoter Activity, and Cis-Elements

The expression patterns of *OsSRABB* were verified using the Rice electronic fluorescent pictograph tool (Rice eFP Browser, https://bar.utoronto.ca/ (accessed on 15 December 2021)) [32], the Rice Expression Profile Database (RiceXPro, https://ricexpro.dna.affrc.go.jp/ (accessed on 15 December 2021)) [33] and the Collections of Rice Expression Profiling (CREP, http://crep.ncpgr.cn/ (accessed on 15 December 2021)) [34]. The promoter activity of P*ssi* was further analyzed by qRT-PCR analysis, details below. Specific primers for expression analysis are listed in Table S1. The *cis*-elements contained in P*ssi* were characterized using the online tool PLACE (https://www.dna.affrc.go.jp/PLACE/ (accessed on 8 December 2021)).

### 4.2. Vector Construction

In order to clone and characterize the promoter of *OsSRABB*, an 858-bp 5′ upstream fragment of *OsSRABB* was amplified as a P*ssi* sequence. The P*ssi* was linked to the *SpCas9* gene [35] by an isothermal recombination reaction-based PCR (IRR-PCR) [36]. Two artificially-designed 20-bp target sequences, “TS1” and “TS2”, with evenly distributed nucleotide differences (A, T, C, G) and moderate GC content (50–55%) (Figure S3) were generated by using the online webtool CRISPR-GE (http://skl.scau.edu.cn/ (accessed on 20 December 2021)) [37]. Then they were fused with the nuclear RNA *OsU3* and *OsU6a* promoters, respectively, to produce two sgRNA expression cassettes. *HPT*, two sgRNA, and the P*ssi*::*Cas9* expression cassettes were inserted between the two pairs of synthetic target sites (TS1 and TS2), generating the T-DNA region, later to be removed. Then the separated *GUS* (β-glucuronidase) encoding sequences, “*GU*” and “*US*”, were linked on both sides of the above T-DNA region and fused into the engineered pCAMBIA1300 to generate pYLP*ssi*::*Cas9* using multi-type plasmid modification based on Gibson cloning [38], in order to test the marker-free excision efficiency of the PssiCHMF system.

### 4.3. Plant Materials and Transformation

All constructs were transformed into rice (*Oryza sativa* L.) variety ‘Zhonghua11’ (ZH11) by Agrobacterium (*Agrobacterium tumefaciens*) strain EHA105-mediated transformation [39]. Root, stem, leaf and sheath samples from wild-type or transgenic rice were collected at the vegetative and reproductive growth periods. The shoot tip, inflorescences, husks, anthers, and pistils were harvested at the reproductive period. Calli were collected at the dedifferentiation period (subcultured for 4 weeks).

### 4.4. qRT-PCR Analysis

Total RNA was separated from each sample using TRIzol regent (Invitrogen, Carlsbad, CA, USA) and the cDNA was synthesized using a cDNA Synthesis SuperMix Kit (TransGen, Beijing, China) according to the manufacturer’s instructions. Gene expression for three replicates was determined using qRT-PCR using SYBR Green qPCR Mix (TransGen, Beijing, China). The *OsUFC1* [40] was utilized as the endogenous control for normalization. The following standard thermal profile was used for qRT-PCR: 95 °C for 3 min; 40 cycles of 95 °C for 10 s; 60 °C for 15 s; 72 °C for 20 s.

### 4.5. GUS Reporter-Aided Analysis

GUS reporter-aided analysis was performed as previously described [41]. Briefly, after the first round of selection (approximately 25 days), transformed calli were immersed in X-gluc staining solution (Real-Times, Beijing, China) at 37 °C in the dark for 16 h. For the pYLP*ssi*::*Cas9* transgenic T_0_ and T_1_ plants, samples of root, stem, leaf, sheath, husk, anther, and pistil were collected during the vegetative and/or reproductive growth stages. After clearing the samples with 75% ethanol to eliminate the background color, samples were observed and photographed under a microscope (Olympus, Tokyo, Japan).

### 4.6. Molecular Characterization of Marker-Free Transgenic Rice

Genomic DNA was extracted from stems of pYLP*ssi*::*Cas9* transgenic T_0_ plants at the reproductive growth stage using the sodium dodecyl sulfate (SDS) method according to Doyle and Doyle [42]. The recombinant and non-recombinant products were amplified in a reaction volume of 20 μL containing 250 nmol L^−1^ of GU-F, T35S-R, and US-R primers. The PCR products were separated on a 0.8% agarose gel. In order to determine the efficiency of the PssiCHMF method, we compared the amount of short and long amplification products. Briefly, if the interval fragment between the two homologous arms had been effectively removed, the GU-F/US-R primers would amplify a 1094-bp “short” product. If the fragment had not been removed, the GU-F/T35S-R primers would amplify a 1277-bp “large” (~12.1 kb product, too large to amplify by GU-F/US-R with short PCR cycles) (Figure 2a).

## Supplementary Materials

The following supporting information can be downloaded at: https://www.mdpi.com/article/10.3390/ijms23031588/s1.
