# Supplementary material for: An Efficient Marker Gene Excision Strategy Based on CRISPR/Cas9-Mediated Homology-Directed Repair in Rice
Source: Int J Mol Sci. 2022 Jan 29;23(3):1588. doi: 10.3390/ijms23031588 (PMC8835944; doi:10.3390/ijms23031588)
Supplement: Supplementary file 1 [file ijms-23-01588-s001.zip › ijms-1554885-supplementary.pdf]

Supplemental information

An efficient marker gene excision strategy based on CRISPR/Cas9-mediated homology-directed repair in rice

Jiantao Tan[1, 2, 3], Yaxi Wang[1, 3], Shuifu Chen[1, 3], Zhansheng Lin[1, 3], Yanchang Zhao[1, 3], Yang Xue[1, 3], Yuyu Luo[1, 3], Yao-Guang Liu[1, 2, 3], Qinlong Zhu[1, 2, 3*]

[1] State Key Laboratory for Conservation and Utilization of Subtropical Agro-Bioresources, South China Agricultural University, Guangzhou 510642, China; tjt@scau.edu.cn (J. T.); wangyx7890@stu.scau.edu.cn (Y. W.); chenshuifu2021@126.com (S. C.); linzhansheng@stu.scau.edu.cn (Z. L.); 18320724774@stu.scau.edu.cn (Y.Z.); xueyang@stu.scau.edu.cn (Y. X.); luoyuyu@stu.scau.edu.cn (Y.L.); ygliu@scau.edu.cn (Y-G. L.); zhuql@scau.edu.cn (Q.Z.)
[2] Guangdong Laboratory for Lingnan Modern Agriculture, Guangzhou 510642, China
[3] College of Life Sciences, South China Agricultural University, Guangzhou 510642, China
* Correspondence: Correspondence: zhuql@scau.edu.cn

Figure S1. *OsSRABB* expresses in several tissues, but no in callus.

Figure S2. *OsSRABB* highly expresses in meristem and inflorescences.

Figure S3. Nucleotide sequences encoding the separated GUS reporter, TS1, TS2 and MCS region sequences.

Figure S4. Detection of the *Cas9* expression cassette in pYLP*ssi*::*Cas9* transgenic plant genome.

Figure S5. CRISPR/Cas9-mediated HDR allows for precise excision and seamless integration of DNA.

Table S1. List of primer used in this study.

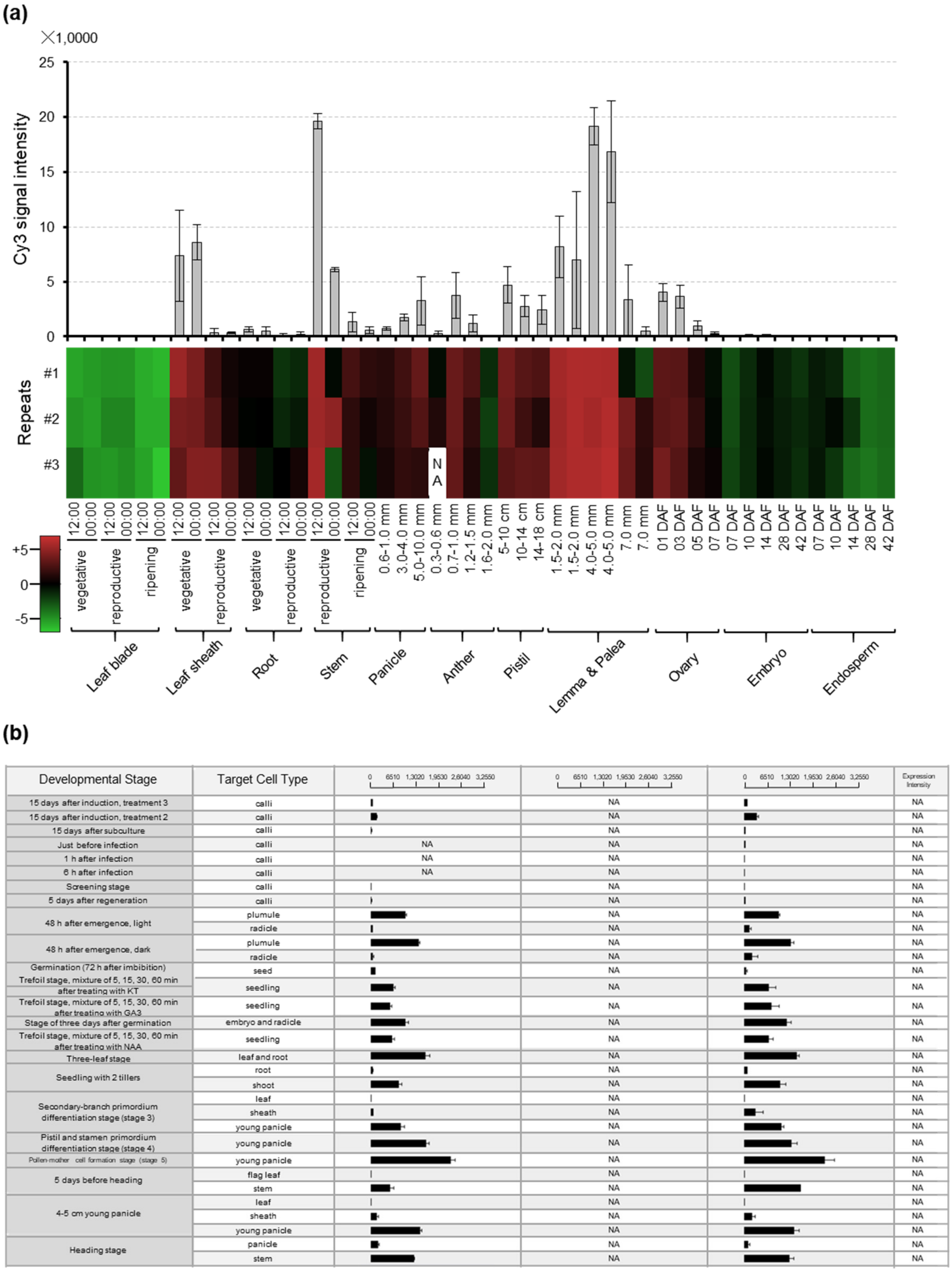

Figure S1. ***OsSRABB*** **expresses in several tissues, but no in callus. (a, b)** Heatmap and ranges of expression of *OsSRABB*. The expression profile for *OsSRABB* in various tissues and organs (including different developmental stages and times of day) is shown as raw data representing the Agilent one-color (Cy3) signal intensity **(a, b)** and normalized data (log2) **(a)** from RiceXpro and CREP. Green indicates low transcript levels, and red indicates high transcript levels. NA, not available.

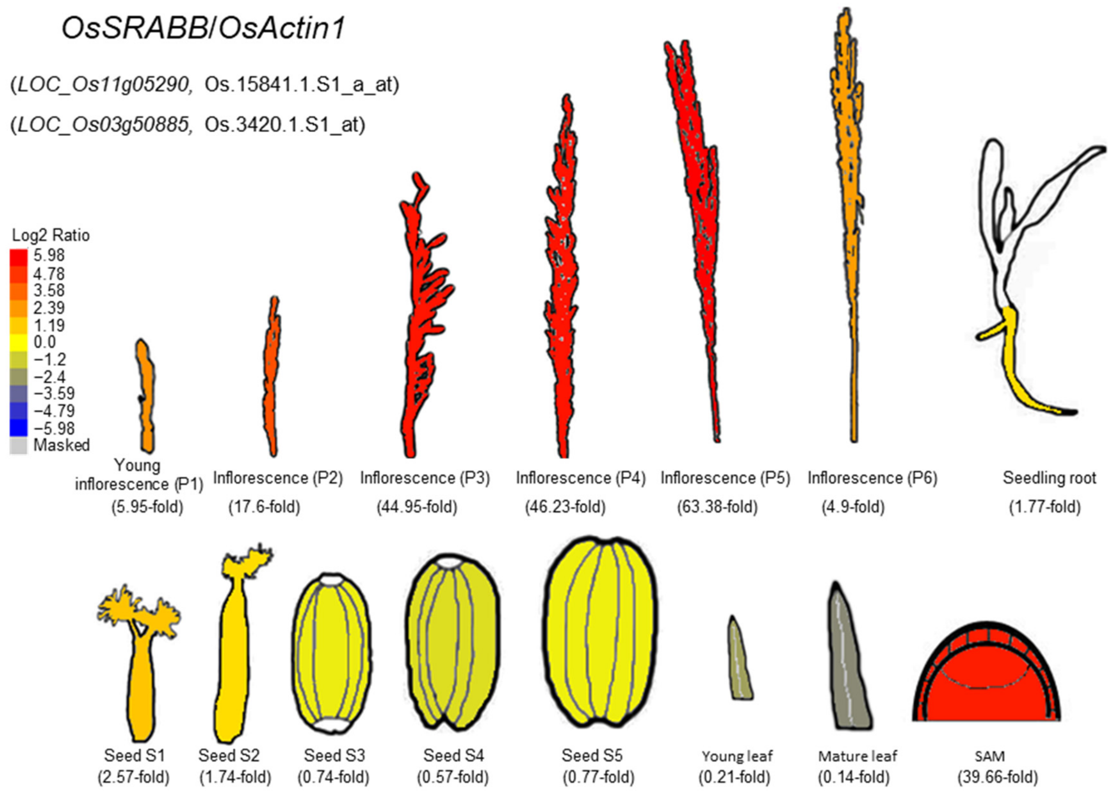

Figure S2. *OsSRABB* highly expresses in meristem and inflorescences. The relative expression levels of *OsSRABB* (*LOC_Os11g05290*) compared with reference gene *OsActin1* (*LOC_Os03g50885*) in meristem (SAM), inflorescences, seeds, leaves and seedling root are shown as normalized data (log2) from Rice eFP Browser. Bule indicates low transcript levels, and red indicates high transcript levels.

Color codes:

G, U, S, TS1, TS2

GUS

ATGGTAGATCTGAGGGTAAATTTCTAGTTTTTCTCCTTCATTTTCTTGGTTAGGACCCTTTTCTCTTTTTATTTTTTTGAGCTTTGA
TCTTTCTTTAAACTGATCTATTTTTTAATTGATTGGTTATGGTGTAAATATTACATAGCTTTAACTGATAATCTGATTACTTTATTT
CGTGTGTCTATGATGATGATGATAGTTACAGAACCGACGAACTAGTCTGTACCCGATCAACACCGAGACCCGTGGCGTCTTCGACCT
CAATGGCGTCTGGAACTTCAAGCTGGACTACGGGAAAGGACTGGAAGAGAAGTGGTACGAAAGCAAGCTGACCGACACTATTAGTAT
GGCCGTCCCAAGCAGTTACAATGACATTGGCGTGACCAAGGAAATCCGCAACCATATCGGATATGTCTGGTACGAACGTGAGTTCAC
GGTGCCGGCCTATCTGAAGGATCAGCGTATCGTGCTCCGCTTCGGCTCTGCAACTCACAAAGCAATTGTCTATGTCAATGGTGAGCT
GGTCGTGGAGCACAAGGGCGGATTCCTGCCATTCGAAGCGGAAATCAACAACTCGCTGCGTGATGGCATGAATCGCGTCACCGTCGC
CGTGGACAACATCCTCGACGATAGCACCCTCCCGGTGGGGCTGTACAGCGAGCGCCACGAAGAGGGCCTCGGAAAAGTCATTCGTAA
CAAGCCGAACTTCGACTTCTTCAACTATGCAGGCCTGCACCGTCCGGTGAAAATCTACACGACCCCGTTTACGTACGTCGAGGACAT
CTCGGTTGTGACCGACTTCAATGGCCCAACCGGGACTGTGACCTATACGGTGGACTTTCAAGGCAAAGCCGAGACCGTGAAAGTGTC
GGTCGTGGATGAGGAAGGCAAAGTGGTCGCAAGCACCGAGGGCCTGAGCGGTAACGTGGAGATTCCGAATGTCATCCTCTGGGAACC
ACTGAACACGTATCTCTACCAGATCAAAGTGGAACTGGTGAACGACGGACTGACCATCGATGTCTATGAAGAGCCGTTCGGCGTGCG
GACCGTGGAAGTCAACGACGGCAAGTTCCTCATCAACAACAAACCGTTCTACTTCAAGGGCTTTGGCAAACATGAGGACACTCCTAT
CAACGGCCGTGGCTTTAACGAAGCGAGCAATGTGATGGATTTCAATATCCTCAAATGGATCGGCGCCAACAGCTTCCGGACCGCACA
CTATCCGTACTCTGAAGAGTTGATGCGTCTTGCGGATCGCGAGGGTCTGGTCGTGATCGACGAGACTCCGGCAGTTGGCGTGCACCT
CAACTTCATGGCCACCACGGGACTCGGCGAAGGCAGCGAGCGCGTCAGTACCTGGGAGAAGATTCGGACGTTTGAGCACCATCAAGA
CGTTCTCCGTGAACTGGTGTCTCGTGACAAGAACCATCCAAGCGTCGTGATGTGGAGCATCGCCAACGAGGCGGCGACTGAGGAAGA
GGGCGCGTACGAGTACTTCAAGCCGTTGGTGGAGCTGACCAAGGAACTCGACCCACAGAAGCGTCCGGTCACGATCGTGCTGTTTGT
GATGGCTACCCCGGAGACGGACAAAGTCGCCGAACTGATTGACGTCATCGCGCTCAATCGCTATAACGGATGGTACTTCGATGGCGG
TGATCTCGAAGCGGCCAAAGTCCATCTCCGCCAGGAATTTCACGCGTGGAACAAGCGTTGCCCAGGAAAGCCGATCATGATCACTGA
GTACGGCGCAGACACCGTTGCGGGCTTTCACGACATTGATCCAGTGATGTTCACCGAGGAATATCAAGTCGAGTACTACCAGGCGAA
CCACGTCGTGTTCGATGAGTTTGAGAACTTCGTGGGTGAGCAAGCGTGGAACTTCGCGGACTTCGCGACCTCTCAGGGCGTGATGCG
CGTCCAAGGAAACAAGAAGGGCGTGTTCACTCGTGACCGCAAGCCGAAGCTCGCCGCGCACGTCTTTCGCGAGCGCTGGACCAACAT
TCCAGATTTCGGCTACAAGAACGCTAGCCATCACCATCACCATCACGTGTGA

TS1 and TS2

CCACCTGGGATTGGAGTCACAGTATGCTACAGACAGTGATAGTGCCACTCACAAGAGG

MCS region

AATTAACTGCAGATCCAGGCCGGCCATAAGCTTTGAGCTCTAAGGCGCGCCTATTTAAATACCTGCAGGT
Asc I *Sbf* I

TTAATTAAGAACGCGTTCAGTTTAAACTTAAATTA
Pac I *Pme* I

Figure S3. Nucleotide sequences encoding the separated GUS reporter, TS1, TS2 and MCS region sequences. The protospacer-adjacent motifs (PAMs) of TS1 and TS2 are indicated in red. The available MCSs are marked by underlines.

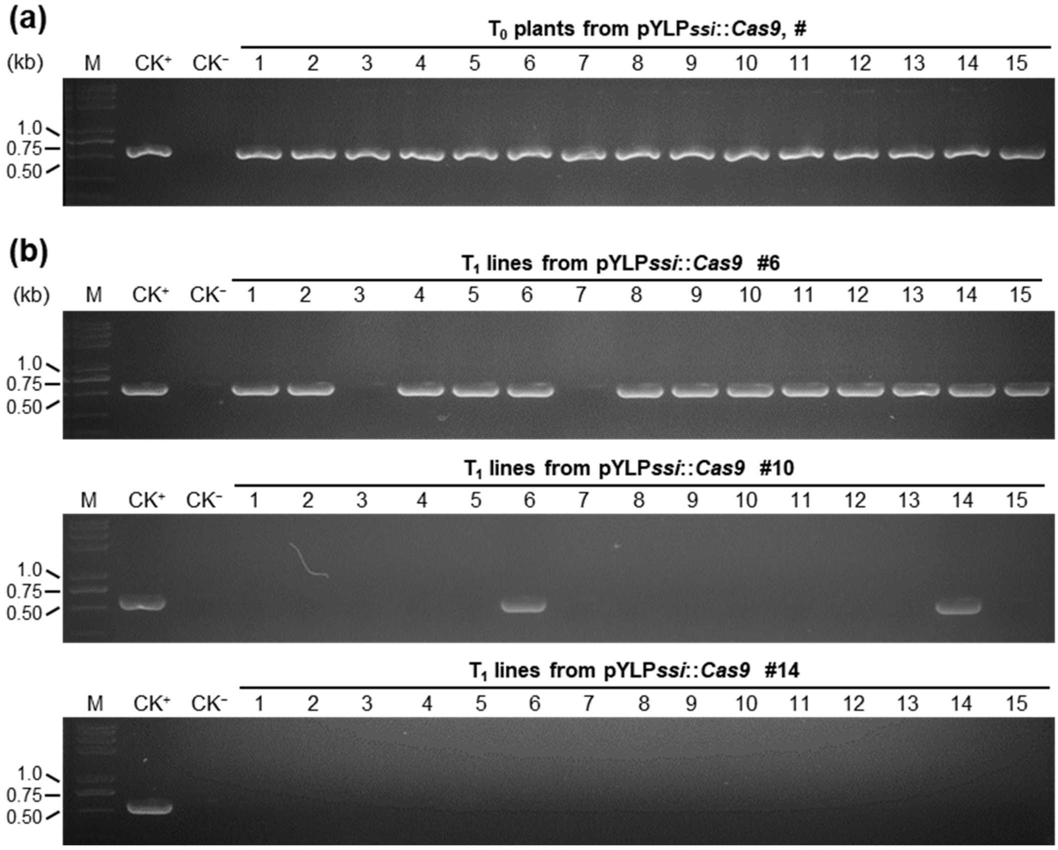

Figure S4. Detection of the *Cas9* expression cassette in pYLP*ssi*::*Cas9* transgenic plant genome. (a, b) PCR using the Cas9-F/Cas9-R primers to detect the *Cas9* expression cassette in pYLP*ssi*::*Cas9* T_0 **(a)** and T_1 **(b)** plants related to Figure 2 and 4. Only when there was still *Cas9* fragment present in the transgenic plant genome, one 608-bp specific product would be generated by Cas9-F/Cas9-R. Otherwise, no any bands would be amplified in the fully excision plants [such as #1 plant (T_1) generated from pYLP*ssi*::*Cas9* #10 (T_0)] and plants without transgene [such as #8 plant (T_1) generated from pYLP*ssi*::*Cas9* #10 (T_0)]. CK^+, the pYLP*ssi*::*Cas9* construct; CK^-, ZH11 plants without T-DNA.

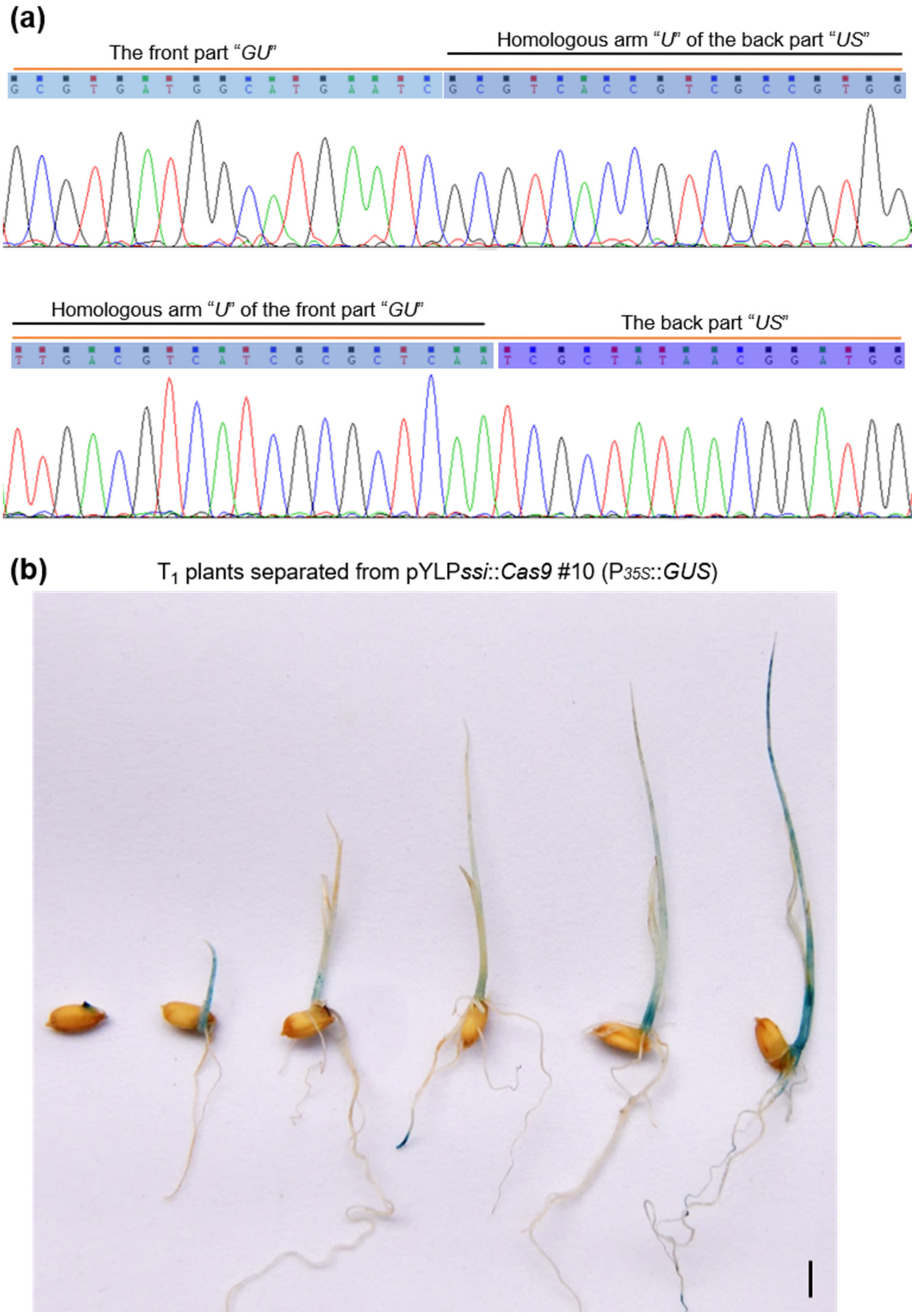

Figure S5. CRISPR/Cas9-mediated HDR allows for precise excision and seamless integration of DNA. (a) Sanger sequencing of the recombinant products amplified from pYLP*ssi*::*Cas9* T_1 lines using GU-F and US-R primers. **(b)** GUS reporter-aided analysis for T_1 seedlings of pYLP*ssi*::*Cas9*#10 line (P_{35S}::*GUS*). Bar = 0.5 cm.

Table S1. List of primer used in this study.

Primers	Sequence (5′-3′)	Purpose
I-1-FU1<br>I-1-R1<br>I-1-R2	ATCCATTCTCAGGCTGTCTCGTCTCGTCTCTTCCCAACAGTTGCGCAGCCTG<br>ACTGTGACTCCAATCCCAGG**TGG**TTGAGCGCGATGACGTCAATC<br>GGCACTATCACTGTCTGTAGCATACTGTGACTCCAATCCCAGG**TGG**	Generating P_{35S} linking "*GU*".
I-2-F1	GTGATAGTGCCACTCACAAG**AGG**TAATTCGGGGGATCTGGATTTTAGTAC<br>GGAGTCACAGTATGCTACAGACAGTGATAGTGCCACTCACAAG**AGG**	Fusing TS1 and TS2

I-2-F2<br>I-2-R	GTTGTCTTACTATTGCTGGCAGGAGGTCAGGCGTATTGGCTAGAGCAGCTTG	to the 5’ end of *HPT* expression cassette.
U-F<br>RU3T1<br>RU6aT2<br>gRNA-R<br>FgT1<br>FgT2	CTCCGTTTTACCTGTGGAATCG<br>CCTGGGATTGGAGTCACAGTGCCACGGATCATCTGCACAACTC<br>CTTGTGAGTGGCACTATCACGGCAGCCAAGCCAGCACC<br>CGGAGGAAAATTCCATCCAC<br>ACTGTGACTCCAATCCCAGGGTTTTAGAGCTAGAAATAGC<br>GTGATAGTGCCACTCACAAGGTTTTAGAGCTAGAAATAGC	Generating *OsU3*::T1-gRNA and *OsU6*::T2-gRNA.
Up-GA-T4<br>gR-GA-T4<br>Up-GA-T5<br>gR--GA-T1	GCCAGCAATAGTAAGACAACACGCAAAGTCGTGGAATCGGCAGCAAAGG<br>CTTGAGTGAGGTTGTAAAGGGAGTTGGCTCCATCCACTCCAAGCTCTTG<br>CCTTTACAACCTCACTCAAGTCCGTTAGAGGTGGAATCGGCAGCAAAGG<br>TGCGTTTGTTTCCGTCTACGAACTCCCAGCCATCCACTCCAAGCTCTTG	Fusing *OsU3*::T1-gRNA and *OsU6*::T2-gRNA to pCAMBIA1300.
II-FU2<br>II-RU3-1<br>II-RU3-2<br>II-RU3-3	GGAAACAAACGCAGAATCCAAGCGCTGCCTTAGTTTCAGTAGAGTTGG<br>TGTCTGTAGCATACTGTGACTCCAATCCCAGG**TGG**CGATCTAGTAACATAGA<br>CAGTGC**CCT**CTTGTGAGTGGCACTATCACTGTCTGTAGCATACTGTGACTCC<br>GTTTCCAGTGCGATTGAGGACCTTCAGTGC**CCT**CTTGTG	Fusing P*ssi*::*SpCas9* expression cassette to the 5’ end of TS1 and TS2.
III-FU3<br>III-RU3	CCTCAATCGCACTGGAAACATCAAGGTCGGCGTCACCGTCGCCGTGGAC<br>GGAGTTGTGGTAATCTATGTATCCTGGTCCCGATCTAGTAACATAGATG	Generating *“US”*.
GUS RT-F<br>GUS RT-R	GGTGAGCAAGCGTGGAACTT<br>TGATGGTGATGGCTAGCGTT	Analyzing the expression of *GUS*.
Pm RT-F<br>Pm RT-R	CGGCGTGATGATGACGACAT<br>CTAAGCTACACCAGCTCAAC	Analyzing the expression of *OsSRABB*.
Cas9 RT-F<br>Cas9 RT-R	CAAGTACTTCGACACCACCATC<br>GGTGGCAGCAGGACGCTTAT	Analyzing the expression of *SpCas9*.
UFC1 RT-F<br>UFC1 RT-R	GATGGCAAGACCCACAAG<br>TCCCGAACCTTGGGCAGT	Analyzing the expression of *OsUFC1*.
GU-F<br>T35S-R<br>US-R	TCAACAACTCGCTGCGTGAT<br>GTCGGGCGTACACAAATCGC<br>CACCGCCATCGAAGTACCAT	Detecting the HDR-mediated marker gene excision.
Cas9-F<br>Cas9-R	TCCAAGTCCAGGCGTCTCGAG<br>AGCTCACCAAGGTGGATCTG	Detecting the *Cas9* expression cassette in plant genome.

Note: The underlines represent the sequences paired with the vector for Gibson cloning. Bold letters represent the PAMs TS1 and TS2.
